# Some unique anatomical scaling relationships among genera in the grass subfamily Pooideae

**DOI:** 10.1093/aobpla/plae059

**Published:** 2024-10-22

**Authors:** Daniel B Spitzer, Troy W Ocheltree, Sean M Gleason

**Affiliations:** Graduate Degree Program in Ecology, Colorado State University, 102 Johnson Hall, Fort Collins, CO 80523-1021, USA; Graduate Degree Program in Ecology, Colorado State University, 102 Johnson Hall, Fort Collins, CO 80523-1021, USA; Department of Forest and Rangeland Stewardship, Colorado State University, 1472 Campus Delivery, Fort Collins, CO 80523-1472, USA; Department of Biological Sciences, Macquarie University, Building E8B, Eastern Road, North Ryde, NSW 2109, Australia; Water Management and Systems Research Unit, United States Department of Agriculture, Agricultural Research Service, 2150 Center Ave, Build D, Suite 320, Fort Collins, CO 80526, USA

**Keywords:** Biological scaling, grass distribution, leaf anatomy, Poaceae, Pooideae, plant hydraulics, xylem

## Abstract

Members of the grass family Poaceae have adapted to a wide range of habitats and disturbance regimes globally. The cellular structure and arrangements of leaves can help explain how plants survive in different climates, but these traits are rarely measured in grasses. Most studies are focussed on individual species or distantly related species within Poaceae. While this focus can reveal broad adaptations, it is also likely to overlook subtle adaptations within more closely related groups (subfamilies, tribes). This study, therefore, investigated the scaling relationships between leaf size, vein length area (VLA) and vessel size in five genera within the subfamily Pooideae. The scaling exponent of the relationship between leaf area and VLA was -0.46 (±0.21), which is consistent with previous studies. In *Poa* and *Elymus*, however, minor vein number and leaf length were uncorrelated, whereas in *Festuca* these traits were positively correlated (slope = 0.82 ± 0.8). These findings suggest there are broad-scale and fine-scale variations in leaf hydraulic traits among grasses. Future studies should consider both narrow and broad phylogenetic gradients.

## Teaser

The cellular structure and arrangements of leaves can help explain how plants survive in different climates, but most studies that analyse these traits are focussed on individual species or distantly related species within Poaceae. While this focus can reveal broad adaptations, it is also likely to overlook subtle adaptations within more closely related groups (subfamilies, tribes). This study, therefore, investigated the scaling relationships between leaf size, vein density and vessel size in five genera within the subfamily Pooideae. The relationship between several leaf anatomical traits was consistent with previous findings. However, several genera exhibited novel anatomical relationships. These findings suggest there is important broad-scale and fine-scale variation in leaf hydraulic traits among grasses. Thus, future studies should consider both narrow and broad phylogenetic gradients.

## Introduction

Grass-dominated ecosystems cover approximately 40 % of all land area on Earth, excluding Antarctica and Greenland (White *et al.* 2000), and grass species constitute some of the most economically important agricultural crops on the planet, accounting for 11.95 tonnes/hectare of annual productivity in wheat, maize and rice alone and over one-third of the total calories consumed by the global human population (FAO 2017). Approximately 4000 of the ~10 000 grass species are found within the Pooideae subfamily, which is the largest subfamily in the Poaceae family. Species in this subfamily are concentrated in temperate climates, but there are many examples of species that occupy hot and dry environments. How this large subfamily diversified and adapted to such a wide range of conditions is largely unknown, although it is clear there are physiological adaptations unique to species growing across this climatic spectrum (Das *et al.* 2021). Identifying the mechanisms that help explain current species distributions will improve our ability to predict changes that are likely to occur under future climate conditions.

The scaling relationship between leaf size, vein length area (VLA) and vessel diameter has provided a mechanistic explanation for species distributions across distantly related species in both eudicot (Sack *et al*. 2012) and monocot plant groups (Baird *et al*. 2021), but these scaling relationships can vary when more closely related species are investigated (Schneider *et al*. 2017). The general pattern of leaf size/anatomy scaling relationships results in species with small leaves having dense vein networks and small-diameter vessels (Price *et al* 2013). During drought and freezing events, air can enter the water column and fill the volume of individual vessels (i.e. embolism), thus blocking the flow of water through the veins. When embolisms do occur, a dense network of veins provides redundancy in the pathway for water movement, such that water can be routed around emboli and continue to supply the most distal reaches of the network with water (Scoffoni *et al.* 2011). It is widely assumed that large diameter vessels are more susceptible to embolism than narrow-diameter vessels, although there is much variation in this relationship across species, and there exists no mechanistic theory to explain the occurrence of embolism under low water potential (Lens *et al*. 2022). In contrast to this, the mechanism linking freezing-induced embolism to vessel diameter is understood and is thought to occur from the release of gas from thawing ice that has formed in the conduit lumen apoplast, with bubble size being limited by lumen diameter (Sperry and Sullivan 1992; Pitterman and Sperry 2003). Although leaves with sparse vein networks (low VLA) and large vessels may be more vulnerable to drought and freezing, utilizing few but large diameter veins (and vessels) provides fast transport of water for a relatively low carbon cost (Verheyden *et al.* 2005; von Arx *et al*. 2012; Bourne *et al.* 2017; Tng *et al.* 2018). Plants with high photosynthetic rates and large leaf canopies tend to utilize this hydraulic architecture to outcompete other species in resource-rich areas (Hacke *et al.* 2022; Olson *et al*. 2023).

The link between vein structure and leaf size has been explained through developmental models, where an understanding of the process of leaf structural development explains the density of veins in the final leaf structure (Sack *et al*. 2012; Baird *et al*. 2021). The major veins of grasses, primary and secondary vein orders, originate at the base of the leaf blade when the leaf primordia is developed and, therefore, are determined early in leaf development. Based on the timing and origin of development, major vein VLA is predicted to decrease with increasing leaf width (LW) since wider leaves cause the major veins to become further spaced apart, although differences in the number of minor veins could partially offset this effect. Minor veins are developed at the tip of the leaf before and as it expands, originating later in time during leaf development. Based on this idea, minor vein VLA is expected to be positively correlated with leaf length (LL) since as leaves expand there is more time and space for new minor veins to form. Among a set of distantly related grass species, these predicted correlations have been shown to occur (Baird *et al*. 2021, 2024). It is interesting that this framework has worked so well since these models are based on the relatively short time scale of leaf development when interspecific differences in anatomy are a result of evolutionary processes that occur on much longer timescales. These patterns, however, emerge across distantly related species and there are examples of leaves deviating from this scaling relationship across more closely related species. For example, among species within a single family, that utilize few vein orders, the relationship between leaf size and VLA deviates from the global relationship (Schneider *et al*. 2017). This is likely because major veins tend to provide the function of higher vein orders in other species. In grasses, many species have few vein orders but we still observe closely related species occupying a wide range of habitats, from tundra to deserts within single genera, so the question is whether more closely related species in this functional group have leaves that follow the global scaling patterns.

The parallel venation of grasses is comprised of longitudinal veins that span much of the LL and are connected transversely through smaller veins (Kuo *et al*. 1972). Although this vein pattern is common among monocotyledons, the implication on plant hydraulic functioning is not well understood. Data on the movement of dye through monocot leaf veins suggest that major veins are responsible for long-distance transport of water and the minor veins are for local distribution (Altus and Canny 1985). This fits with the developmental model of veins in grasses, where the major veins originate first and in the basal meristem of the leaf and so have the potential to span the length of the leaf, while minor veins originate more acropetally as the cells expands and the leaf widens, and so may only be capable of localized water distribution. However, as plants evolve and adapt to environmental conditions, it is unclear how quickly/easily plants might adjust the number of veins in a certain vein order compared to adapting to other physiological mechanisms. As adaptation results from random mutations, it is just as feasible that physiological adaptations to different environments would occur instead of structural adaptations, which might be observed in closely related species. Certainly, physiological adjustments, at some point, would require structural adjustments and would explain the global scaling relationship described above. However, among more closely related species, it is unclear if the same scaling relationship between VLA, vessel diameter and leaf size is maintained as plants adapt to different conditions.

To evaluate whether scaling relationships established across distantly related species can be applied across more closely related species, we focus on the relationships between anatomy, leaf size, and leaf gas exchange of multiple species within five genera constrained to a single subfamily of grasses and propose the following hypotheses: (i) the diameter of vessels will scale isometrically with the diameter of vascular bundles for minor and major veins; (2) vessel diameter will be inversely correlated with VLA; (3) the density of the major veins will be negatively correlated with LW, but (4) vessel diameter and minor vein VLA will scale more closely with LL than LW. Finally, (5) photosynthesis will be positively correlated with VLA and the average size of vessels. To that end, anatomical traits were measured from greenhouse-grown species of grasses from the subfamily Pooideae. These data were also analysed for relationships between anatomy and climate traits. Based on the previous body of research reporting correlations between leaf anatomy and climate variables (Blackman *et al*. 2010; Sack and Scoffoni 2013; Baird *et al*. 2021), we hypothesized that there would be strong relationships between leaf vasculature and climate variables, especially between precipitation and xylem characteristics.

## Materials and Methods

### Plant material selection and germination

Five species were selected from each of five genera: *Poa, Hesperostipa, Elymus, Festuca* and *Bromus* (See Table 1). The species selected in this study are all phylogenetically classified as part of the Pooideae subfamily; this was done to ensure that all species were separated by relatively recent evolutionary divergences. Species were also selected such that their habitats spanned a wide range of temperature and precipitation across North America. Plants in this study were grown from seeds provided by the United States Department of Agriculture Germplasm Resource Information Network. Unfortunately, not all species germinated regardless of any pre-treatments we attempted and so we were not able to measure five species in all genera. Specimens used for gas exchange measurements were grown during the summer of 2016 in 35-cm-length Deepots (D60 series, Stuewe and Sons, Inc, Tangent, USA) while the remaining specimens were grown in 10-cm-length ‘cone’-tainers (Ray Leach Cells 3, Stuewe and Sons, Inc, Tangent, USA), with a 70–30 % mix of potting soil (Promix HP, Quakertown, USA) and fritted clay (Greens’ Grade Porous Ceramic Topdressing, Buffalo Grove, USA), respectively. This substrate was fertilized with slow-release fertilizer (Osmocote Plus, Scotts Miracle-Gro Company, Marysville, USA) at a ratio of 10 mL fertilizer L^-1^ soil substrate. The soil substrate was saturated with water before seeds were planted, and specimens were misted until the full expansion of their fourth leaf, then watered three times per week.

**Table 1.** List of the species germinated for data collection.

Genus	Specific epithet	Genus	Specific epithet	Genus	Specific epithet
*Bromus*	*anomalus*	*Festuca*	*altaica*	*Hesperostipa*	*neomexicana*
*Bromus*	*inermis*	*Festuca*	*arizonica*	*Poa*	*alpina*
*Bromus*	*laevipes*	*Festuca*	*californica*	*Poa*	*arida*
*Elymus*	*canadensis*	*Festuca*	*idahoensis*	*Poa*	*compressa*
*Elymus*	*elymoides*	*Festuca*	*roemeri*	*Poa*	*fendleriana*
*Elymus*	*hysterix*	*Festuca*	*rubra*	*Poa*	*glauca*
*Elymus*	*lanceolatus*	*Hesperostipa*	*comata*	*Poa*	*secunda*

### Anatomical analysis

Whole-leaf samples (base to tip of lamina) were collected from five randomly selected individuals of each species for the purpose of anatomical examination. The third or fourth full leaf was harvested from each plant after full expansion. Samples were stored in a solution of formalin, acetic acid, ethanol and deionized water (formalin-acetic acid-alcohol fixative solution). Anatomical samples were collected halfway along the lamina length of each specimen, using a razor to cut a cross-sectional sample ~0.5 mm in thickness. The distance between the cross-section and the leaf tip was also recorded. Each cross-sectional sample was stained using safranin-o and fast green. Microscopic images of vein anatomy were taken of half the total lamina width using a ZEISS Axio Scope.A1 in conjunction with ZEN microscope software (Carl Zeiss Microscopy, Germany). Images were measured using Fiji open-source image analysis software. The number of vein orders of each species was quantified, and 1° and 2° veins were classified as ‘major’ veins, and 3° and 4° (if present) were classified as ‘minor’ veins. We defined major veins as having at least two of the three following characteristics: (1) vein was at least 50 % larger than the smallest vein, (2) vein had bundle sheath extension, (3) vein had at least two xylary vessel elements (i.e. metaxylem lacuna) 100 % larger than remaining xylary vessel elements. Although this criteria differs slightly from what others have used to define vein orders (Baird *et al*. 2021), it was developed to help us objectively assign vein orders since leaf veins do not always fall neatly into the orders defined previously. However, we are confident that what we defined as ‘major’ is consistent with previous research. The anatomical traits of up to five major veins and minor veins per leaf were measured, but all veins were counted and classified into a vein order. Vein length density was calculated as total vein length per unit area (cm/cm^2^) by measuring the width of the leaf and then counting the number of veins in the leaf, then multiplying by the length of the leaf. The diameter of the vein including the bundle sheath (μm) was measured as the distance from the outer edge of the bundle sheath to the opposite outer edge. Vein diameter (*D*_vein_, μm) was measured from the innermost edge of the bundle sheath to the opposite inner edge. Diameter of the bundle sheath cells (*D*_BS_) was then estimated by subtracting the vein diameter measured from the innermost edge from the vein diameter measured from the outermost edge of the bundle sheath. The diameters of vessel elements (*D*_vessel_, μm) were measured as the distance between the inner edge of the vessel element cell wall to the opposite inner edge. Vessel element wall thickness (WT; μm) was also measured. ‘MAJ’ is added to each subscript when the data reported were measured on major veins and ‘min’ is added to the subscript when the data represent the minor veins.

### Climate envelope analysis

Species distribution data were collected from the Global Biodiversity Information Facility (GBIF). Climate data were obtained from weather stations closest to the location where the seed was harvested as reported by GBIF. In addition to weather station observations, gridded and interpolated climate data were retrieved from WorldClim at 0.1° resolution. For each reported occurrence of each species in the GBIF database, climate data from the closet grid point was retrieved and added to the data set. If two reported occurrences were equally close to the same grid point, that point was only used once to avoid pseudo-replication. Once this data set was generated for each species, the 5th, 50th and 95th percentiles of each climate variable (see list of variables in supplement) were calculated and used to define the climate envelope of each species. Because we hypothesized that hydraulic architecture would relate to the temperature and precipitation of the climate of origin, we focussed our investigation on climate variables that would capture these abiotic stressors: mean annual precipitation (MAP), mean annual temperature (MAT), the temperature of the wettest and driest quarters and MAP of the warmest quarter.

### Statistical analysis

Scaling relationships were evaluated using the ‘sma’ function in the ‘smatr’ package to account for variability in both *x* and *y* variables. In cases where we expected variation between the *x* and *y* variables to be explained by a power function, we log10-transformed both axes. This included relationships between leaf dimensions (width and length) and vessel number (Price *et al*. 2007; Gleason *et al*. 2018), and between leaf dimensions (i.e. pathlength ~ LL) and conduit diameter (Anfodillo *et al*. 2006). Axes were also log10-transformed in cases where we wanted to test an expected linear or proportional relationship between *x* and *y* variables, for example, the relationship between conduit diameter and cell WT at a given buckling pressure (Brodribb and Holbrook 2005), as well as conduit diameter (or conduit number) between different vein orders (Gleason *et al*. 2018). Variables were first tested for differences between genera by including ‘genus’ in a model to test for differences between the coefficients—slope (hereafter ‘scaling coefficient’) and intercept. If no differences were found, then a single scaling relationship was used and reported. If differences were found, then the genera with unique coefficients were removed and analyses were performed individually on each group.

## Results

### Leaf size and vein characteristics

Leaf width and LL explained 89 % of the variation in leaf area with only 11 % remaining that was from measurement error and differences in leaf taper **[see Supporting Information—****Fig. S1****]**. The VLA of the major veins was negatively correlated with the leaf area and had a slope ^-^0.51 ± 0.12 **[see Supporting Information—****Fig. S2B****]**. Across all genera, we found a positive linear slope between VLA of the minor veins and leaf area, but this pattern was driven by an increase in minor vein VLA among narrow leaves and a decrease in minor vein VLA among wider leaves [**see Supporting Information—****Fig. S2A**]. Since the majority of species with small leaves were within *Festuca*, we fit a regression for this genus separate from the other genera. The positive slope between leaf area and VLA for *Festuca* explained 50 % of the variation in VLA (*P* = 0.10), and the negative slope for the other genera explained 48 % of the variation in VLA among the other genera (*P* < 0.01).

**Figure 1.**
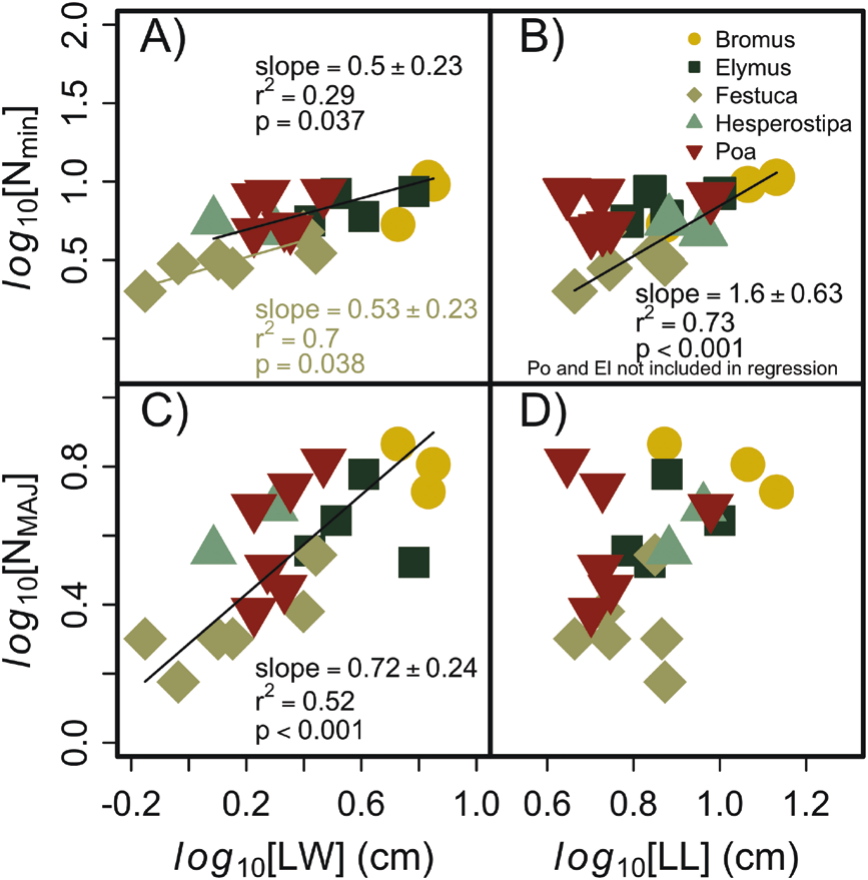
Relationship between leaf dimensions and number of minor and major veins. LW was highly correlated with the number of minor (A) and major (C) veins but not all genera shared a common slope. LL was also correlated with minor (B) and major (D) veins, but the correlations were weaker for this leaf dimension. The dashed line represents a 1:1 reference line.

Since leaf area was included in the calculation of VLA (resulting in auto-correlation) and we were interested in the different scaling relationships between LL and LW, we relied more on the relationship between vein number and leaf dimensions than VLA in our analyses. The scaling coefficient between vein number and leaf dimensions differed between some of the grass genera we sampled, and LW correlated more strongly with vein number (Fig. 1A and C) than LL (Fig. 1B and D). All genera had the same slope and intercept between LW and *N*_MAJ_ veins, but *Festuca* had a unique intercept from the other genera for *N*_min_ versus LW, meaning that *Festuca* had fewer minor veins per unit LW compared to the other genera. The slope of the LW × *N*_MAJ_ veins was <1 (0.72), indicating that the density of major veins decreases with LW. The slope of the LW × *N*_min_ was ~0.5 for all genera and did not differ significantly from the major veins. There was no correlation between LL and *N*_MAJ_ veins, but there was a positive correlation between LL and *N*_min_ veins for *Festuca*, *Bromus* and *Hesperostipa*; *Poa* and *Elymus* exhibited no change in *N*_min_ veins with changing LL. The slope of the *N*_min_ ~ LL regression for *Festuca*, *Bromus* and *Hesperostipa* was not different from 1, suggesting that the number of minor veins changed in proportion to changes in LL.

**Figure 2.**
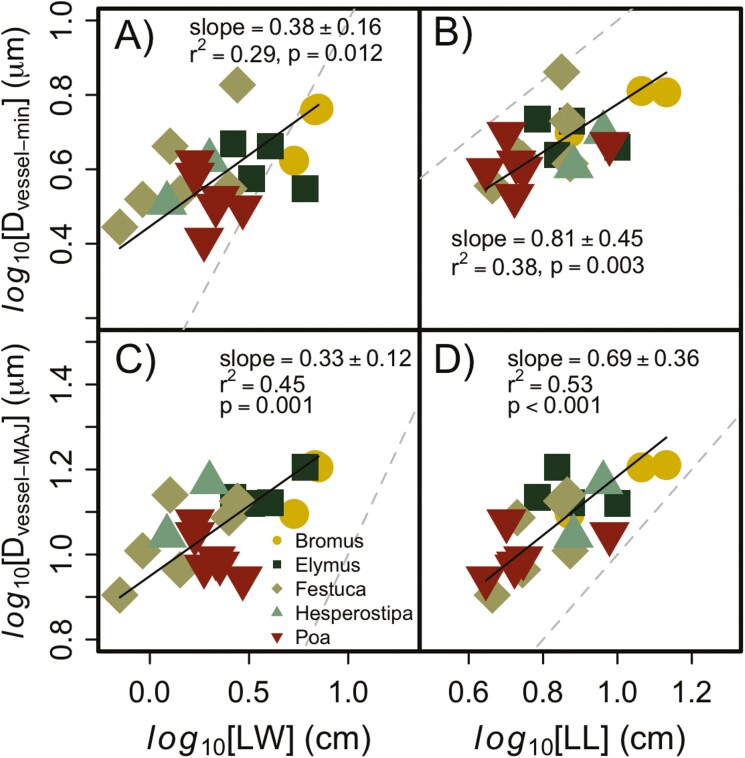
Relationship between leaf dimensions and the average diameter of vessel elements found in major (*D*_vessel-MAJ_) and minor veins (*D*_vessel-min_). The grey dashed line represents a 1:1 relationship between the variables. The scaling relationship between LW and vessels in minor (A) and major (C) veins had a slope of 1, but the slope of the scaling coefficient between LL and these same variables (B and D) both were greater than 1. The dashed line represents a 1:1 reference line.

Average lumen diameter was positively correlated with leaf area [**see Supporting Information—****Fig. S2**] and the individual components of leaf size (Fig. 2). LL explained ~10 % more of the variation in vessel diameter (*D*_vessel_) than LW for the major veins, but LL explained more of the variation than LW for minor veins. Wider and longer leaves had veins with larger vessel diameters, on average, but vessel diameter scaled similarly with LL and LW among all genera. However, wider *Poa* leaves tended to have slightly smaller *D*_vessel_ (minor and major veins) than narrow leaves, although the regression for this single genus was not significant for either scenario (*P* > 0.2). For LW, the scaling coefficient with *D*_vessel-MAJ_ and *D*_vessel-min_ were both <1, so these variables change in proportion to each other across the species and genera we studied. For LL, the scaling coefficient was not different than 1 for the minor veins (0.81 ± 0.45) or the major veins (0.69 ± 0.35).

### Scaling of vein characteristics

**Figure 3.**
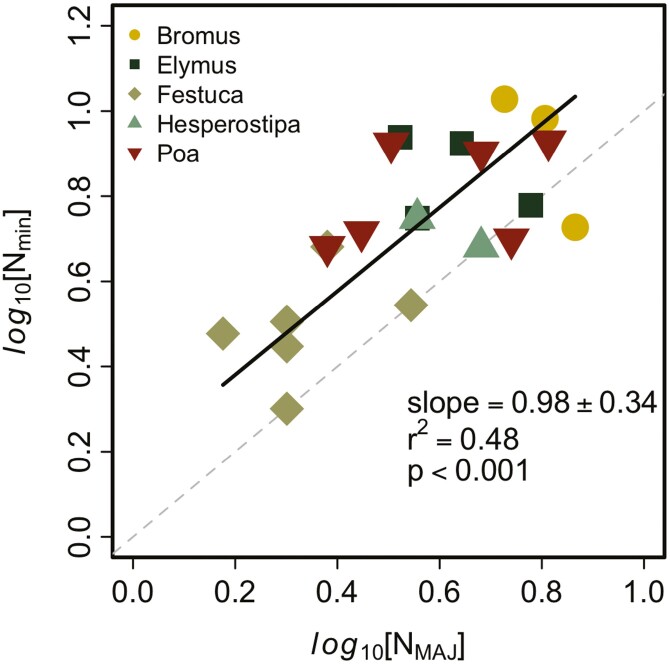
Scaling relationship between the number of major versus minor veins (*N*_MAJ_ and *N*_min_, respectively) among the selected grass species. The dashed line represents a 1:1 reference line.

The number of minor veins scaled to the number of major veins with a scaling coefficient of 1 for all genera except Festuca (Fig. 3). A scaling coefficient of 1 suggests that the ratio of major:minor veins remained constant across species, although the number of minor veins was always greater than major veins except in *Festuca* (intercept 0.48 ± 0.18). For genera with at least five species, we found no significant correlation between the number of major and minor veins.

**Figure 4.**
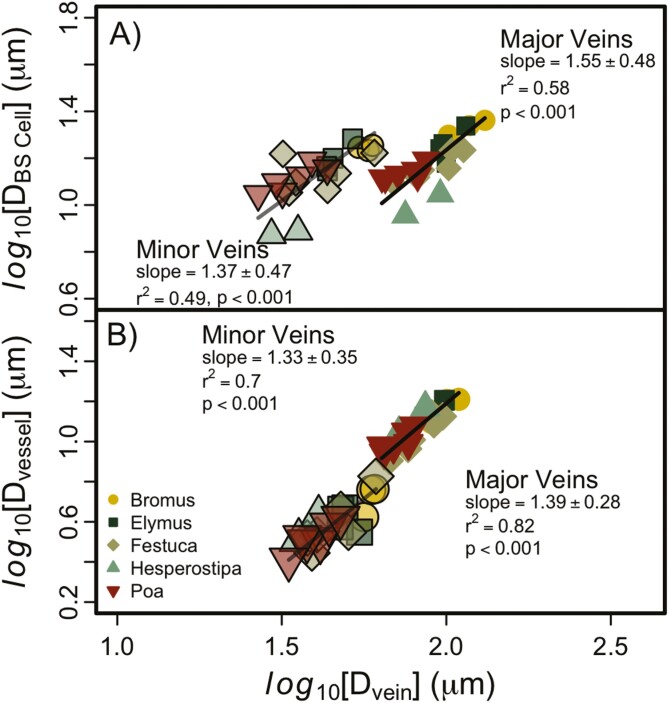
Relationship between vein diameter (*D*_vein_) and vessel diameter (*D*_vessel_, panel ‘A’), and between *D*_vein_ and bundle sheath cell size (*D*_BSCell_, panel ‘B’). There were no differences between the genera, and only the intercept of the relationship between *D*_vein_ and *D*_BSCell_ differed.

The size of the xylem and BS cells scaled closely with the size of the vein in which they were found (Fig. 4). The diameter of BS cells (*D*_BS_) in both major and minor veins had a scaling coefficient of ~1 with *D*_vein_ and, interestingly, the average size of BS cells did not differ significantly between the two vein orders; major veins had diameter of 15.64 µm ± 3.69 and minor veins had 13.90 µm ± 3.16. The diameter of vessels, however, was smaller in minor veins than in major veins. The scaling coefficient between *D*_vein_ and *D*_vessel-minor_ was not statistically different than 1 (1.33 ± 0.35) but was >1 for the major veins (1.39 ± 0.28). Both vein orders had identical slopes and intercepts, so there appears to be strong coordination between vein and vessel size across all vein orders among this set of grass species.

**Figure 5.**
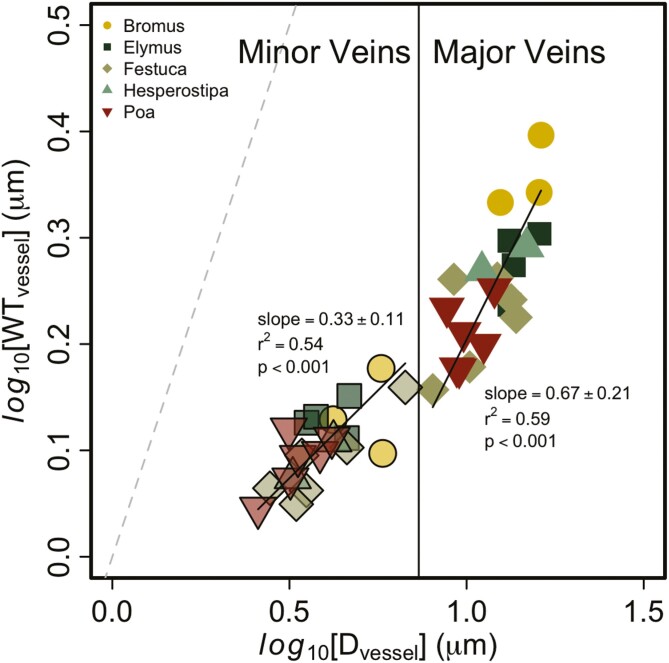
Relationship between the average cell wall thickness (WT_vessel_) and the lumen diameter of vessel elements (*D*_vessel_) in the minor (left side) and major veins (right side). The major veins had a steeper slope (0.66 ± 0.23) compared to the minor veins (0.33 ± 0.12). However, there were no differences between genera in the relationship between WT and lumen diameter within each vein order. The dashed line represents a 1:1 reference line.

The ratio of cell WT to lumen diameter (*D*_vessel_) of vessel elements provides information on the resistance of these cells to buckling under tension (Fig. 5). The scaling coefficient between these two variables was <1 for both vein orders, but the major veins had a slightly larger scaling coefficient (0.66 ± 0.23) than the minor veins (0.33 ± 0.12). These small scaling coefficients are indicative of smaller vessels having relatively thicker cell walls, but the change in WT with *D*_vessel_ is greater in the major veins, so the WT:*D*_vessel_ ratio of these cells remained similar across vessel sizes.

### Anatomical versus gas exchange and climate

**Figure 6.**
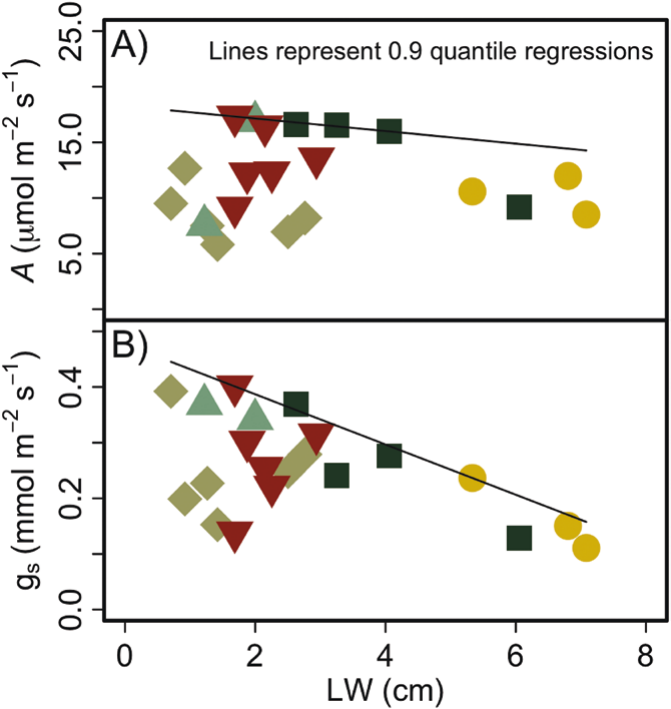
Relationship between LW and photosynthesis (panel A) and stomatal conductance (panel B). Regression lines shown fit through the 90th percentile of the gas exchange data. The most striking pattern is the lack of species with wide leaves having fast rates of stomatal conductance.

Neither leaf size nor vein traits correlated with gas exchange or climate across all genera investigated (data not shown). However, there was evidence of an upper threshold between LW and the gas exchange variables we measured (Fig. 6). A percentile regression through the 90th percentile highlights the low *g*_*s*_ of wide leaves and a wider range of *g*_*s*_ among narrow-leaved grasses (Fig. 6B). The variability below the 0.90 percentile regression line is mainly a result of Festuca and Poa because a regression with only *Bromus*, *Elymus* and *Hesperostipa* is significant (*P* = 0.004) and 80 % of the variability in *g*_*s*_ was explained by LW for these three genera. Although there was a similar pattern for *A* (Fig. 6A), the slope of the percentile regression is not as steep and, therefore, a threshold is not as clear for this parameter.

**Figure 7.**
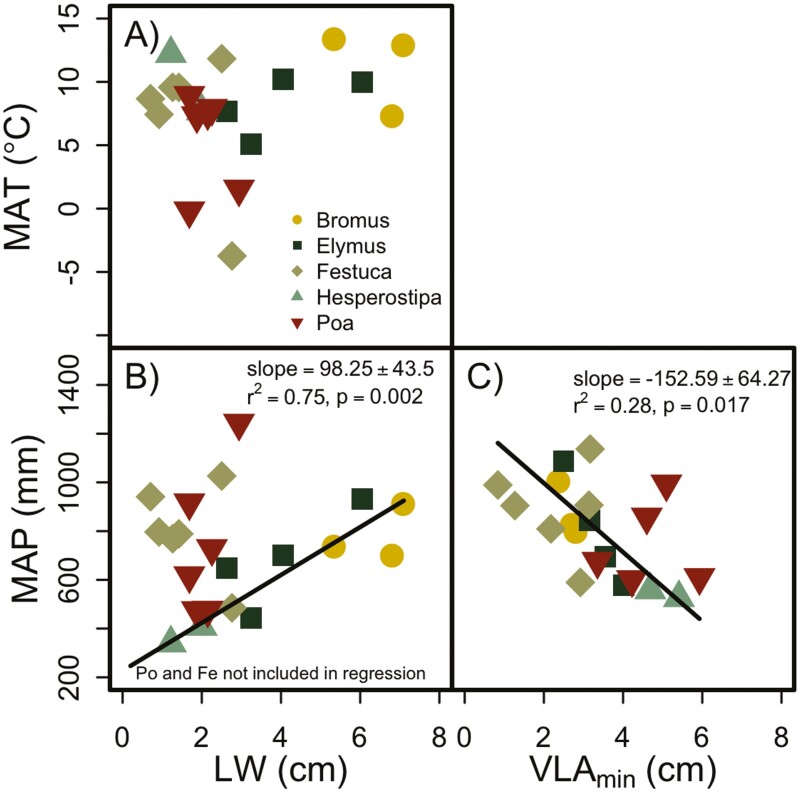
Correlation between LW and VLA of minor veins (VLA_min_) and the climate of origin for each species. No clear patterns emerge between LW and MAT (panel A) and MAP (panel B), but when *Poa* and *Festuca* are excluded, narrow-leaved species appear to be found in drier areas compared to wider-leaved species (panels A and C). Species with high VLA of the minor veins were found in drier habitats than species with low VLA_min_ (panel C).

There were very few relationships between climate and the leaf characteristics we measured. VLA_min_ was the only anatomical trait that correlated with climate, and there was a negative correlation with MAP (Fig. 7). There were no clear correlations between leaf dimensions and MAP or MAT (Fig. 7) across all genera. However, there was a correlation between LW and MAP among the species in the *Bromus*, *Elymus* and *Hesperostipa* genera (Fig. 7C). *Poa* and *Festuca* had a large range of variability in MAP where the species could occur but relatively small variability in LW. There was also an obvious absence of plants with wide leaves from cold climates (Fig. 7A).

## Discussion

The vascular system within leaves represents a large investment by the plant for the transport of water and photosynthate and, because of this investment, leaf venation tends to be built to maximize transport but minimize cost to the leaf lamina (McCulloh *et al*. 2003), which results in interspecific scaling relationships between leaf size and hydraulic architecture. However, studies supporting the idea of global scaling relationships have largely focussed on two areas: (1) leaves of woody eudicots, and (2) comparisons across large phylogenetic gradients with very few representatives from a single genus. We wanted to explore whether the scaling relationships that have emerged across genera and families are the same relationships that have emerged from speciation as subfamilies and genera diversified by using non-woody angiosperms as the focal functional group. There is evidence, specifically focussed on hydraulic traits, of closely related species deviating from the global scaling pattern, which likely emerges because of the unique distribution of vein orders in the Ochnaceae family (Schneider *et al*. 2017). Another example of a disconnect between broad phylogenetic relationships and relationships among closely related species comes from a widely used relationship between leaf hydraulic conductance and photosynthetic rates (Brodribb *et al.* 2007). Across broad phylogenetic scales, there is a positive relationship between these variables, but if one focuses only on the angiosperms there appears to be very little relationship between these variables. We are not suggesting one scale of investigation is more important than another, just that both need to be evaluated to fully understand the evolution patterns that exist to inform the potential changes that may occur in the future. Although the majority of scaling relationships we investigated were consistent with previously reported scaling relationships and were consistent across all genera we measured (i.e. same slope and intercept), there were important scaling relationships that diverged from global scaling patterns for some of the genera. Our species selected from *Festuca* and *Poa*, which are the two largest genera in the grass family, showed unique scaling relationships among some of the variables we investigated. If these relationships apply to the rest of the members of these genera, this is the first report we are aware of to show unique scaling relationships within individual genera in a single subfamily and suggests we need to investigate patterns of vascular adaptation at multiple phylogenetic scales to better understand the patterns we observe in nature.

Before we discuss our results in more detail, it is important to consider the different roles minor and major veins might serve in monocot versus eudicot leaves. In bifurcating networks, like the veins of most eudicot leaves, smaller veins are in series with larger veins but occur further ‘downstream’ along the water transport pathway. Small veins in grasses, in contrast, run in parallel with large veins and are only connected through the few and small transverse veins. Altus and Canny (1985) suggested that the large veins provide long-distance transport and the smaller veins provide localized transport of water, which makes sense due to the smaller diameter vessels found in minor veins (Fig. 4). In addition to providing water transport, these veins are also responsible for collecting and transporting photosynthate but, unfortunately, the role of different vein orders on photosynthate transport has not been quantified. In the following discussion of our results, we will assume the minor veins serve as localized transport of water and the large veins provide long-distance transport; we will revisit their potential roles in photosynthate transport as well.

### Divergent scaling between leaf size and VLA

The size (and specifically the area) of grass leaves is determined by LW, length and the tapering of the leaf; in our data set LW and length explained 89 % of the variability in area, suggesting the tapering function is relatively constant across the species we measured. Since these dimensions independently explained such a large amount of the variability in leaf area, we were able to investigate the impact of width and length separately on vein characteristics, which is important since developmental theory suggests these dimensions will correlate with different vein characteristics (Baird *et al.* 2021). The number of major veins in a leaf correlated most strongly with LW, but a combined slope of <1 for all genera indicates that wider leaves tend to have a lower density of major veins among these genera, which is consistent with the global pattern of decreasing VLA of primary and secondary veins in eudicot leaves (Sack *et al*. 2012) and grass leaves (Baird *et al*. 2021). Furthermore, the confidence intervals for the slope between the leaf area and VLA [**see Supporting Information–****Fig. S2A**] include the slope calculated by Sack *et al*. (2012) and are consistent with findings from Baird *et al*. (2021), suggesting that the grasses we measured follow this same global relationship. There was no correlation between leaf length and the number of major veins (primary and secondary), which also agrees with the developmental scaling theory.

Based on the developmental scaling theory, we would expect minor vein VLA to correlate better (and positively) with LL than LW. The Baird *et al.* developmental model (2021) predicts a greater probability for minor vein primordia to arise from longer leaves than shorter leaves because longer leaves require longer developmental times. However, we did not find that to always be true for the species we measured. *Poa* and *Elymus* exhibited no change in the number of minor veins with differences in LL, but the other genera showed a strong positive correlation between these variables (*r*^2^ = 0.73). We do not have an explanation for why *Poa* and *Elymus* would differ from the other genera, but this does suggest that either the development of leaf veins differs among some genera in the grass family or the role of different vein orders may vary between species/genera requiring different densities of minor veins. *Festuca* had fewer minor veins per LW compared to the other genera (intercept of 0.30 vs. 0.46), but all genera had a similar slope of ~0.5 (Fig. 2). The small slope suggests that, like major veins, the number of minor veins decreases per unit width in wider leaves; however, the emergent pattern was not so clear. *Festuca* actually exhibited increasing minor vein VLA with increasing leaf area and the other genera exhibited a decline in minor vein VLA. In general, *Festuca* had the smallest leaves and, along with *Poa*, some of the narrowest leaves. We somewhat subjectively separated *Festuca* from the other genera, but the relationship could also be interpreted as small leaves versus large leaves, and so it is unclear whether *Festuca* has evolved a different strategy in utilizing different vein orders or whether there is some biophysical difference between small and large leaves that would cause the relationship of VLA with leaf area to switch. The unique scaling relationships for minor veins in *Festuca, Poa* and *Elymus* highlight the importance of investigating evolutionary patterns across broad phylogenetic gradients *and* within closely related groups.

Despite the unique scaling relationships we identified in *Poa*, *Elymus* and *Festuca* for minor veins, the proportion of major:minor veins was consistent across all genera. The scaling coefficient between the number of minor and major veins is not different than 1 (Fig. 3), which suggests the proportion of these two vein classes does not change across species or leaf sizes. There were generally more minor veins than major veins, but the offset of this scaling relationship did not differ from zero. Although there was a lot of scatter in this relationship, it does suggest some optimal relationship between the number of minor versus major veins in either/both the transport of water and collection/transport of photosynthate.

Unlike vein number, vessel diameter in both major and minor veins correlated better with LL than LW, although the differences were small. Longer leaves present a longer path for water to move within leaves and could cause significant resistance to water movement. To minimize the resistance of path length, the diameter of xylem cells widens from the tips of grass leaves to the base (Ocheltree and Gleason 2024). A similar tapering occurs in the woody tissue of trees, and the consistency between species results in a strong correlation between tree height and the diameter of vessels at the base of the tree (West *et al.* 1999; Olson *et al.* 2021). The scaling coefficient between plant height and vessel diameter is very close to 0.2 in the woody tissue of angiosperm and gymnosperm trees (Anfodillo *et al*. 2006; Olson *et al*. 2021), but we found a scaling coefficient of 0.69 between LL and vessel diameter measured at the mid-point of each leaf. The larger exponent we found here is likely because one key assumption in models of vessel tapering developed for trees is that the network of ‘pipes’ is not leaky to water, but the ‘pipes’ in our case, the veins in leaves, are definitely leaking as they supply water for cell hydration and transpiration along the length of the leaf (Ocheltree and Gleason 2024). Despite the difference in the scaling of vessel diameter and path length between grass leaves and woody tissue, our results suggest longer and wider grass leaves are utilizing larger-diameter vessels to transport water, which may make them more susceptible to drought and freezing-induced embolism formation (Davis *et al*. 1999; Lens *et al*. 2022).

### Leaf characteristics and climate

As we have discussed, large diameter vessels are generally more susceptible to embolism formation during drought and freezing events, and, based on this information, we expected grass species that rely on large diameter vessels for water transport to be unable to occur in hot/dry and cold areas. However, this pattern did not emerge in our data, for example, there was no correlation between either MAP or MAT and the average diameter of vessels in leaves (Fig. 4B). The density of minor veins (VLA_min_) was negatively correlated with MAP, however, which is consistent with previous data in eudicot leaves (Sack and Scoffoni 2013). In eudicot leaves, the high VLA might provide redundancy in the pathway for water transport to cells in the leaf (Sack *et al*. 2012). However, in grass leaves, it is not clear that the parallel veins would be able to provide the same redundancy. Transverse veins that connect parallel veins typically have a single small vessel (Chonan *et al.* 2011), so whether this structure could provide the same redundancy as the branching pattern of eudicot leaves is unclear. Even though we did not find a correlation between vessel diameter and climate, the minor veins do have smaller-diameter vessels than the major veins, so perhaps vessel diameter still plays a role in grass distribution. Assuming small-diameter vessels do provide resistance to embolism formation, then a plant with dense minor veins might be able to continue to transport water during drought and after freezing temperatures even if vessels in the major veins have embolized. Although this may sound like a plausible theory, the vein orders first exhibiting embolism formation is not consistent among grasses (Canny 2001; Johnson *et al.* 2018; Ocheltree *et al*. 2020). So, this leaves an unanswered question—if it is true that high VLA confers drought tolerance in grass leaves, what is the mechanism?

Since VLA_min_ density was correlated with LW, it is not surprising that we found a correlation between LW with MAP, but the correlation only existed when *Festuca* and *Poa* were excluded from the regression. There was a small range of LWs for these two genera, but they spanned a large range of environmental conditions based on MAP. LW did not show a correlation with MAT, but there is clearly an absence of wide-leafed species in cold–wet climates, which is consistent with the effects of leaf size and boundary layer on species distributions in both grasses (Baird *et al*. 2021) and eudicots (Wright *et al*. 2017). The poor correlation between hydraulic architecture and climate could be because herbaceous perennials, like the grasses studied here, are known to survive harsh environmental conditions by senescing their leaves and resprouting from buds that are positioned at, or below, the soil surface. With this strategy, a grass plant could be vulnerable to embolism formation but still occur in a relatively dry or cold climate by simply growing when conditions are suitable or in a microsite that is suitable for growth.

### Leaf size and physiology

Although anatomy was a poor predictor of distribution for the species we studied, leaf size was negatively correlated with photosynthesis and stomatal conductance. An upper boundary exists between these metrics, such that species with wide and long leaves do not achieve high rates of photosynthesis or stomatal conductance, although this is less clear for photosynthesis. Similar threshold relationships exist in the grass family, with wide-leaved grasses closing their stomata at relatively higher xylem water potentials (*Ψ*_crit_; higher values indicating low drought tolerance), while narrow-leaved grasses exhibit a wide range of *Ψ*_crit_ values (Craine *et al*. 2013). The lower *g*_*s*_ of wide-leaved species could contribute to higher leaf temperatures in warmer areas if latent/sensible heat flux was meaningfully reduced via lower rates of transpiration and thicker boundary layers (Cowan 1972; Wright *et al*. 2017). And so our results, combined with those of *Ψ*_crit_ (Craine *et al*. 2013), would suggest that it would be more challenging for larger-leaved grasses to grow during hot and dry conditions because of their low stomatal conductance and thick leaf boundary layers. But they would also be excluded from growing during cold periods due to their large diameter vessels and the susceptibility of these vessels to freezing-induced embolism formation.

### Scaling of vein and cell size

The size of different cell types associated with vascular bundles and water transport scale closely across a range of species in a single subfamily of grass and align with scaling relationships found among eudicots. For example, the largest-diameter veins house larger-diameter vessel elements and are surrounded by large bundle sheath cells. The scaling relationships among these variables all had a slope of ~1 and did not differ among the different genera. Furthermore, this scaling was consistent across both the major and minor veins. We have now added a large set of grass species to the dataset on the coordination of cell size within plant leaves, and the fact our results agree with previous research on angiosperms (Brodribb *et al*. 2013; John *et al*. 2013; Sack and Scoffoni 2013; Baird *et al*. 2024) suggests that this phenomenon is consistent across all C3 angiosperms and reinforces the idea that there is either coordinated growth among the cells that contribute to transport in leaves or some constraint that would result in the size of these cells being linked together during growth and development. We specify C3 angiosperms because the hydraulic architecture of C4 plants suggests that these relationships can be decoupled at some scales since C4 plants have larger bundle sheath cells than C3 but smaller diameter vessel elements (Kocacinar and Sage 2003).

Interestingly, although bundle sheath size exhibited a different intercept (*D*_BS_ ~ *D*_vein_) between major versus minor veins, the absolute values of bundle sheath size did not differ significantly between these vein orders (Fig. 4). If the role of the minor veins was *only* to provide localized water transport (i.e. do not contribute to mechanical stability or other metabolic functions), why would the bundle sheath cells be the same size in minor and major veins? This question suggests a role for the minor veins beyond just water transport. The role of bundle sheath cells is still an active area of research, but these cells may play a role in the movement of water from xylem to mesophyll cells, and in C3 plants they are capable of photosynthesis and other metabolic processes (Leegood 2008). It seems likely that the bundle sheath cells of the minor veins would contribute to the loading of photosynthate into the phloem, and the size of these cells in minor veins suggests an equal role compared to major veins. These results highlight the need to assess the role of major versus minor veins from a metabolic and photosynthate transport perspective rather than only through the lens of water transport (Altus and Canny 1982).

Despite the uniform scaling of vein and vessel diameter across vein orders, the ratio of WT to lumen diameter in vessel (*D*_vessel_) elements did differ between these vein orders. For both vein orders, the slope was <1, indicating that smaller vessels have thicker cell walls proportionate to the diameter of their lumen than larger vessels. This would provide them more resistance to buckling (Zhang *et al.* 2016) when water tensions are great and could provide greater tolerance to drought (Blackman *et al*. 2010; Lens *et al*. 2022; Olson *et al*. 2023). The slope in minor veins (0.33) was lower than in major veins (0.66), which suggests that *D*_vessel_ in major veins increases more per change in cell WT than those in minor veins, which may be because these veins are responsible for long-distance water transport and increasing the lumen diameter to reduce hydraulic resistance may be more important in the major versus minor veins. Embolisms have been observed to occur in the major veins first in wheat (Johnson *et al.* 2018), which is in the same subfamily as the species we studied, and in maize (Canny 2001), with embolisms only appearing at greater water tensions in the minor veins, which aligns with our results. However, embolism appeared first in the minor veins of bamboo (Ocheltree *et al*. 2020), which does not align with the anatomy we found in this subfamily, but bamboo is not closely related to the species we measured, so the anatomical patterns may differ among subfamilies/tribes within the grass family.

## Conclusion

When we investigate relationships among leaf size, anatomy, and climate, there are interesting and unique patterns that emerge among species within individual genera that are missed when we only investigate distantly related species, and both scales of inquiry and adaptation are important to understand. For example, comparing distantly related species may reveal ‘jumps’ in adaptions that have driven broad patterns in distribution and function we observe at the landscape scale, but investigating more intensely within groups of closely related species can also reveal important differences between species that would otherwise be missed. As we try to understand what controls the current distribution of species to predict future changes to these distributions, it will be important to understand the adaptation at both these phylogenetic scales. Furthermore, teasing apart the roles of different vein sizes will be an important next step in understanding the structure and function of grass leaves. Previous work has focussed on the differences in water transport between large and small veins, but an understanding of the role these veins play in the accumulation and transport of photosynthate is currently lacking. Finally, we need to understand why there is no strong correlation between the mean annual climate metrics and leaf size, VLA and vessel diameter. It is possible that a large fraction of variation in leaf and vein traits is associated with species-specific growth phenology and the climates associated with active periods of growth, rather than mean annual climate summaries. Although this has not yet been tested, including phenology and growth season climate metrics may improve our ability to explain the current distributions of grass species, as well as predict their future distributions.

## Supporting Information

The following additional information is available in the online version of this article –

**Figure S1.** The figure showing the relationship between LW and LL.

**Figure S2.** The figure showing the relationship between leaf area and vein density in both major and minor veins.

**Figure S3.** The figure showing the relationship between the leaf area and the vessel diameter of both major and minor veins.

**Table S1.** A list of climate variables collected from weather stations and used in the analysis.

**Table S2.** A summary table of phylogenetically controlled analysis of the traits included in the primary figures.

plae059_suppl_Supplementary_Material

## Data Availability

Relevant data used for the analyses in this article can be found via Dryad at: https://doi.org/10.5061/dryad.547d7wmhs
