# Supplementary material for: Some unique anatomical scaling relationships among genera in the grass subfamily Pooideae
Source: AoB Plants. 2024 Oct 22;16(6):plae059. doi: 10.1093/aobpla/plae059 (PMC11538577; doi:10.1093/aobpla/plae059)
Supplement: plae059_suppl_Supplementary_Material [file plae059_suppl_supplementary_material.pdf]

SUPPLEMENTAL FIGURES AND TABLES

	Leaf Area = Leaf Width + Leaf Length		
	p-value	Partial r^2	Total r^2
Leaf Width	2.12 e-5	0.72	0.89
Leaf Length	3.9 e-5	0.17	

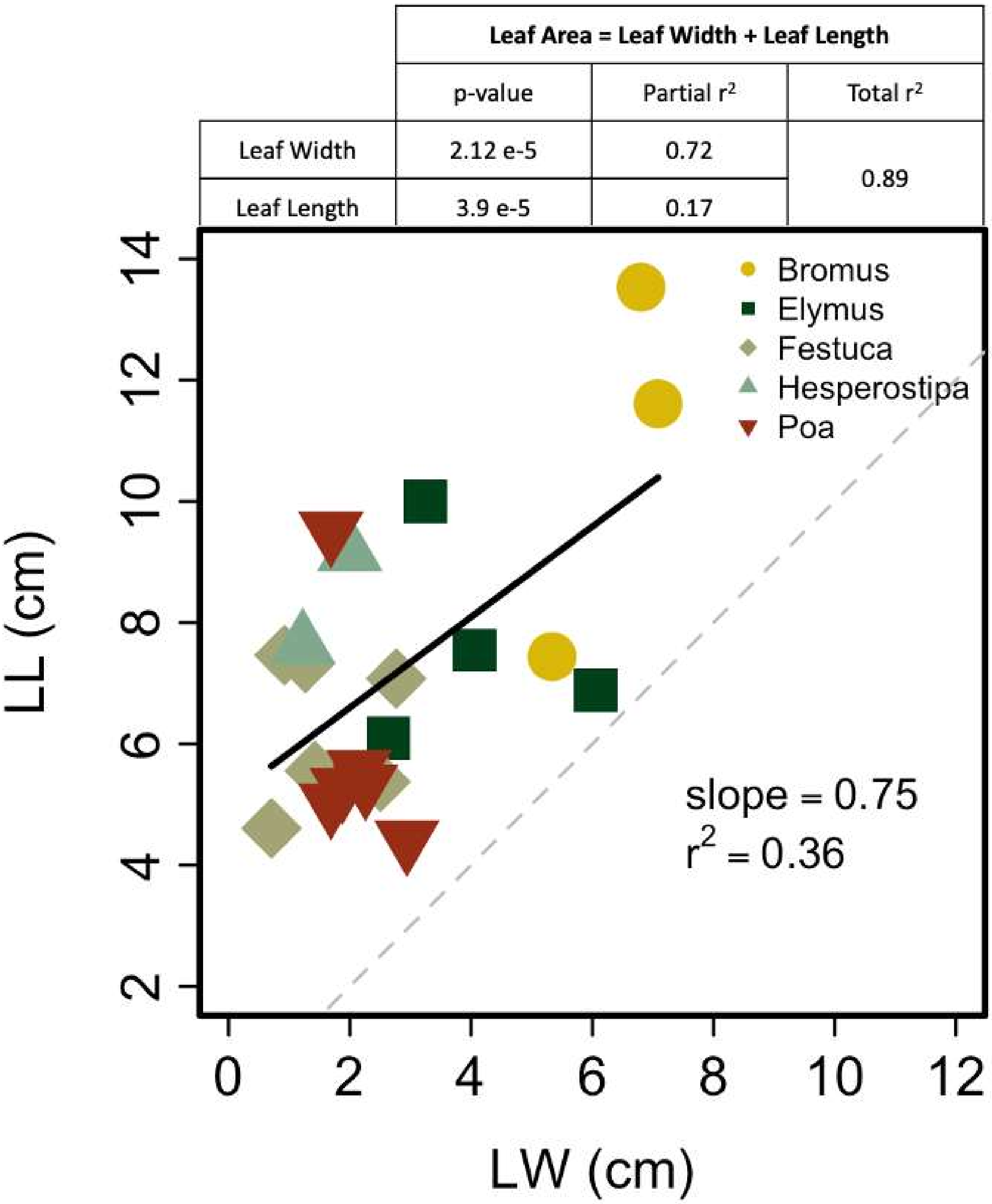

Figure S1. Correlation between Leaf Width (LW) and Leaf Length (LL) along with the partial r2 for each variable in the model explaining leaf area (table at top). LW explains the greatest amount of the variability in leaf area and, together with LL, 89% of the variability in leaf area is explained.

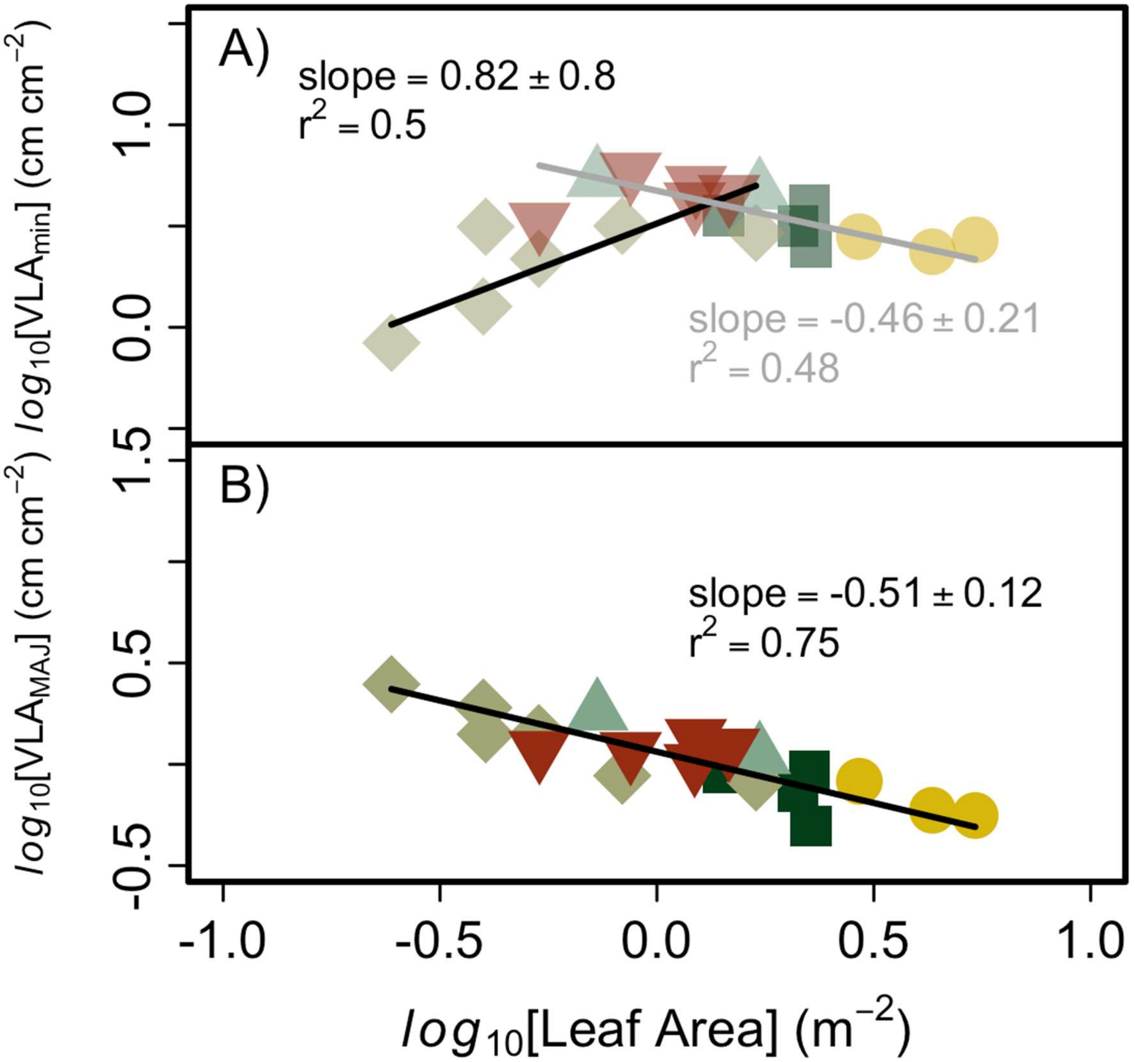

Figure S2. Scaling relationship between $\log_{10}$ of leaf area and $\log_{10}$ of vein density in the minor veins (VLA_{min}, panel A) and major veins ($VLA_{MAJ,}$ panel B). The density of major veins scales consistently across all genera (lower panel), but minor vein density of the *Festuca* species had a positive correlation with leaf area, but the other genera had a negative correlation. The negative scaling coefficient of the VLA_{min} was not significantly different than the global scaling coefficient found in Baird et al. 2021.

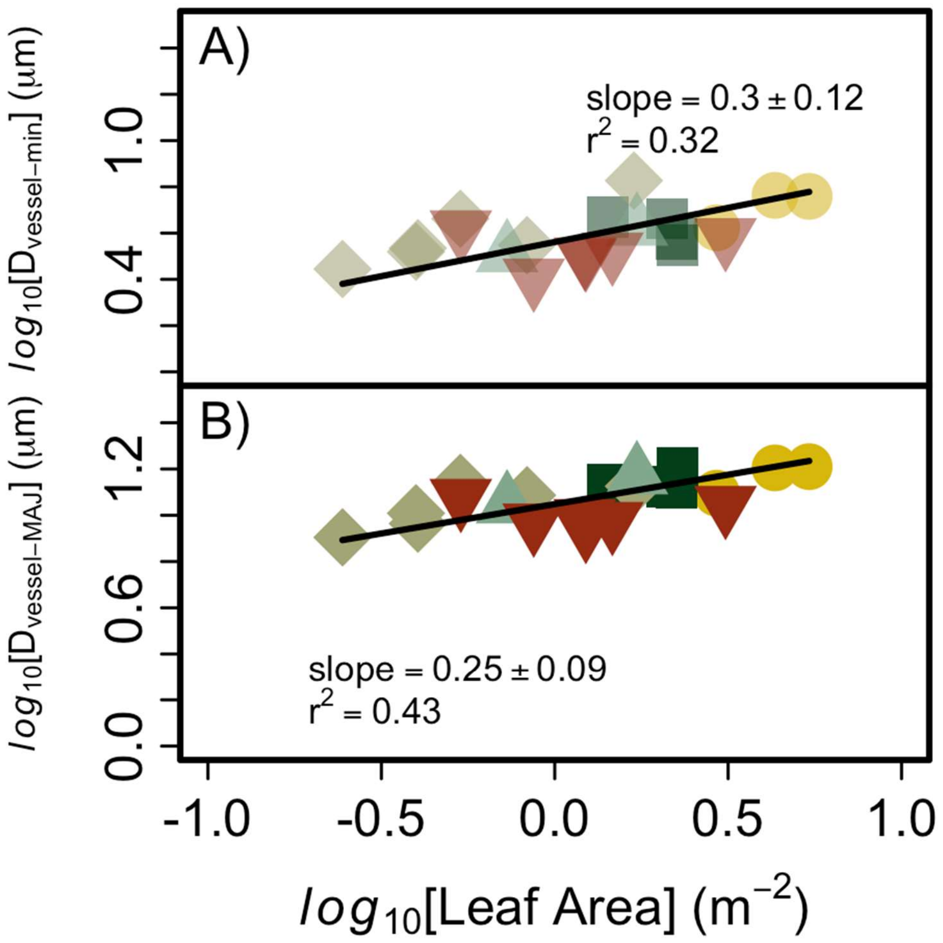

Figure S3. Scaling relationships between $\log_{10}$ of leaf area (panel B) and the lumen diameter of vessels in the minor ($D_{vessel\text{-}min}$, panel A) and major ($D_{vessel\text{-}MAJ}$, panel B) veins. Although the absolute values of $D_{vessel\text{-}min}$ was smaller than $D_{vessel\text{-}MAJ}$, the scaling coefficients were the same for vessels in both vein classes.

Climate Variables	
**Precipitation Variables**	**Temperature Variables**
MAP	MAT
Precipitation in the wettest month	Mean diurnal temperature
Precipitation in the driest month	Isothermality
Precipitation seasonality	Temperature seasonality
Precipitation during the wettest quarter	Maximum temperature during warmest month
Precipitation during the driest quarter	Minimum temperature during coldest month
Precipitation during the warmest quarter	Annual temperature range
Precipitation during the coldest quarter	Mean temperature during wettest quarter
	Mean temperature during driest quarter
	Mean temperature for warmest quarter
	Mean temperature for driest quarter

Table S1. A list of climate variables collected from weather stations. Note that MAT = Mean annual temperature, MAP = Mean annual precipitation. Data generated were then calculated into 5th, 50th, and 95th percentiles.

	**Included variables**		**Phylogenetically controlled**			**Uncontrolled**		
	x-axis	y-axis	Slope	r2	p-value	Slope	r2	p-value
**Figure 1**	Leaf Width	N minor (noFe)	1.49	0.25	0.065	0.5	0.29	0.037
		N major	1.07	<0.01	0.808	0.72	0.52	<0.001
	Leaf Length	N minor (no Poa or El)	0.94	0.72	0.002	1.61	0.73	<0.001
		N major	-0.85	<0.01	0.74	1.51	0.16	0.072
**Figure 2**	Leaf Width	D minor	0.94	0.05	0.352	0.38	0.29	0.011
		D major	1.54	0.14	0.105	0.33	0.45	<0.001
	Leaf Length	D minor	0.85	0.03	0.468	0.81	0.4	0.003
		D major	1.21	0.047	0.358	0.69	0.53	<0.001
**Figure 3**	N major	N minor	1.08	0.02	0.559	0.98	0.48	<0.001
**Figure 4**	Dvein*maj	Dvessel*maj	0.81	0.75	<0.001	1.39	0.82	<0.001
	Dvein*min	Dvessel*min	0.63	0.93	<0.001	1.33	0.7	<0.001
	Dvein*maj	BS*maj	0.91	0.57	<0.001	1.55	0.58	<0.001
	Dvein*min	BS*min	0.88	0.28	0.016	1.37	0.29	<0.001
**Figure 5**	Wtvessel*maj	Dvessel*maj	0.39	0.36	0.005	0.67	0.59	<0.001
	Wtvessel*min	Dvessel*min	0.37	0.55	<0.001	0.33	0.54	<0.001
**Figure 6**	NA							
**Figure 7**	Leaf Width	MAT	1.37	0.25	0.026	2.21	0.05	0.327
	Leaf Width	MAP	2.6	0.03	0.466	120.78	0.04	0.407
	VLAmin	MAP	-228.22	0.15	0.089	-152.59	0.28	0.017

Table S2. A summary table of phylogenetically controlled analysis of the traits included in the primary figures. Note also the inclusion of summary statistics for analysis that was not phylogenetically controlled.
